# Cancers in Northern Thailand

**DOI:** 10.2349/biij.4.3.e46

**Published:** 2008-07-01

**Authors:** P Kamnerdsupaphon, S Srisukho, Y Sumitsawan, V Lorvidhaya, V Sukthomya

**Affiliations:** 1 Division of Therapeutic Radiology and Oncology, Faculty of Medicine, Chiang Mai University, Thailand; 2 Department of Surgery, Faculty of Medicine, Chiang Mai University, Thailand; 3 Department of Otolaryngology, Faculty of Medicine, Chiang Mai University, Thailand

## Abstract

A retrospective study was undertaken to assess cancers in northern Thailand using the Chiang Mai Cancer registry and Maharaj Nakorn Chiang Mai Hospital records from January 2001 to December 2005. Maharaj Nakorn Chiang Mai Hospital is the university hospital for the Faculty of Medicine, Chiang Mai University. There were 4,108 new cancer cases being treated at the institution. The distribution of patients were (a) 32% from Chiang Mai, (b) 42% from nearby provinces of Lampoon, Phayao, and Chiang Rai, (c) 20.4% from other northern provinces, and (d) 1.2% from other parts of Thailand. Based on the data, the most common cancers by relative frequency are cancers of the lung, cervix, liver, breast, and non-Hodgkin's lymphoma. The current treatment options used to manage these most common cancers are described in this article.

## INTRODUCTION

Cancer is one of the major public health problems in Thailand. In 2002, cancer became the third common cause of death in the Northern part of Thailand [1]. A retrospective analysis was undertaken to assess the cancers in Northern Thailand. The sources for this data analysis were the Chiang Mai Cancer Registry and Maharaj Nakorn Chiang Mai Hospital, using the medical records of patients from January 2001 to December 2005. Maharaj Nakorn Chiang Mai Hospital is the university hospital of the Faculty of Medicine, Chiang Mai University. The hospital has 1,800 beds and serves about 415,000 out-patients and 49,200 in-patients each year.

## MATERIALS AND METHODS

In 2005, there were 4,108 cases of new invasive cancer at Maharaj Nakorn Chiang Mai Hospital [2]. Thirty-six percent were Chiang Mai residents, 42.0% came from nearby provinces (Lampoon, Lampang, Phayao and Chiang Rai), 20.4% came from the other provinces in the northern region, and only 1.2% resided outside the northern part of Thailand (Table 1).

**Table 1:** Locations of the invasive cancer cases.

**Location**	**cases**	**%**
**NORTHERN REGION**	**4042**	**98.4**
Chiang Mai	1479	36.0
Chiang Rai	648	15.8
Lampoon	503	12.2
Phayao	375	9.1
Lampang	198	4.8
Nan	197	4.8
Phrae	175	4.3
Mae Hong Son	160	3.9
Tak	108	2.6
Sukhothai	77	1.9
Uttaradit	57	1.4
Kamphaingphet	19	0.5
Phitsanuloak	16	0.4
Phichit	11	0.3
Phetchabun	10	0.2
Nakhon Sawan	6	0.1
Uthai Thani	3	0.1
**CENTRAL REGION**	**36**	**0.9**
**NORTH-EASTERN REGION**	**8**	**0.2**
**SOUTHERN REGION**	**4**	**0.1**
**FOREIGNERS**	**19**	**0.5**
**TOTAL**	**4108**	**100.0**

### Age and sex

There were 1,810 male and 2,298 female cancer cases in the year 2005, with a male-to-female ratio of 1:1.3, but 1,135 (49.4%) of the cancers in females occurred in sex-specific sites (i.e. breast and reproductive organs) while only 80 cases (4.4%) of sex-specific cancers (i.e. prostate, testis, and penis cancers) occurred in males. When sex-specific sites were excluded, the male-to-female ratio increased to 1.5:1 [2].

**Figure 1:**
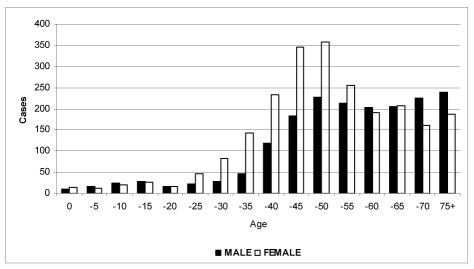
Age distribution of new cancer cases in Maharaj Nakorn Chiang Mai Hospital, 2005.

Ages ranged from below one year to 98 years. The median age at diagnosis was 55 years, 57.3 years for males and 59 years for females. In the age group of 25 to 59, there were more female cancer patients than males, while in the age group above 60, there were more male cancer patients than female cancer patients (Fig. 1). There were 100 cases of cancer in children (below age 15), accounting for only 2.4% of all cases, but there were 1,618 cases in the old-age group (age 60 and above), accounting for 39.4% of all cases [2].

There were 214 in situ patients and they were not included in this analysis. Uterine cervical cancer in situ was the most common, accounting for 63.6% of cases.

### Stage of disease

Twenty eight percent were diagnosed at an advanced stage (20.3% distant metastasis and 8.3% regional node metastasis), and 55.9% were diagnosed at a localised stage and locally advanced. In 835 cases of distant metastasis, 13.2% had multiple sites of metastasis. The most common site of distant metastasis was lung (21.7%), followed by distant lymph nodes (21.4%), liver (16.5%), bone (14.0%), and brain (12.6%).

### Leading sites of cancer cases

Of the invasive cancer in both sexes combined, lung cancer was the most common (14.1%), followed by cervix, liver, breast, and non-Hodgkin’s lymphoma (Table 2). Together these five types of cancer accounted for 51.2% of all new cancers. For males, the most common cancer was lung cancer, accounting for 19.8% of all new cases, followed by liver cancer, non-Hodgkin’s lymphoma, nasopharyngeal cancer and rectal cancer. For females, the most common cancers were cervix cancer, accounting for 23.7% of all new cases, followed by breast, lung, ovary, and liver cancer.

**Table 2:** The 10 leading malignancies in Maharaj Nakorn Chiang Mai Hospital, 2005.

	**Males**	**cases**	**%**		**Females**	**cases**	**%**		**Both sexes**	**cases**	**%**
1	Lung	359	19.8		Cervix	544	23.7		Lung	578	14.1
2	Liver	319	17.6		Breast	324	14.1		Cervix	544	13.2
3	NHL	114	6.3		Lung	219	9.5		Liver	433	10.5
4	Nasopharynx	77	4.0		Ovary	143	6.2		Breast	333	8.1
5	Bladder	72	4.0		Liver	115	5.0		NHL	217	5.3
6	Rectum	61	3.4		NHL	103	4.5		Ovary	143	3.5
7	Colon	60	3.3		Corpus	94	4.1		Colon	119	2.9
8	Stomach	57	3.1		Thyroid	74	3.2		Rectum	115	2.8
9	Leukaemia	52	2.9		Colon	59	2.6		Nasopharynx	107	2.6
10	Prostate	48	2.7		Rectum	54	2.3		Thyroid	100	2.4

### Childhood cancer

Approximately 24 percent of Thailand's population is younger than 15 years old [3]. At Maharaj Nakorn Chiang Mai Hospital, there were 100 cases of childhood cancers (ages below 1 to 14), accounting for 2.4% of all cancer cases in 2005. The most common childhood cancer was leukaemia, accounting for 49.0% of childhood cancers, followed by brain and nervous system (13.0%), NHL (6.0%), bone (5.0%), and eye (5.0%). Leukaemia, brain and nervous system were the common causes of death in childhood cancers.

### Primary Treatment

In 2004, there were 18.8% of cancer patients who received symptomatic treatment alone due to advanced disease or patient refusal. The other 81.2% received definitive treatment [1]. The majority of the patients received single modality treatment and the most common primary treatment was surgery, followed by chemotherapy, and radiation therapy (Table 3).

**Table 3:** Type of Primary Treatment for Cancers in 2004.

**Type of Treatment**	**Cases**	**%**
**Single Modality**	**2,176**	**77.6**
Surgery	1,208	43.1
Chemotherapy	553	19.7
Radiotherapy	414	14.8
Others	1	0.0
**Combined modalities**	**628**	**22.4**
Surgery + Chemotherapy	332	11.8
Surgery + Radiotherapy	137	4.9
Radiotherapy + Chemotherapy	107	3.8
Surgery + Radiotherapy + Chemotherapy	47	1.7
Others	5	0.2
**Total**	**2,804**	**100.0**

## DISCUSSION

This retrospective study shows that lung cancer and cancer of the cervix are the two most common cancers in Northern Thailand. A comprehensive report on the characteristics, diagnosis and staging, and treatment is presented in the management of these diseases.

### Lung cancer

**Figure 2:**
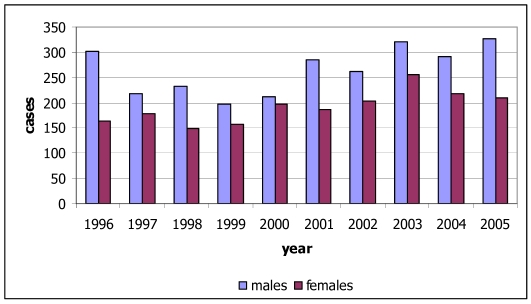
Number of new cases of lung cancer by sex, 1996-2005.

**Figure 3:**
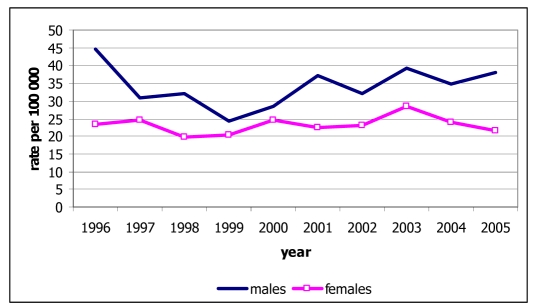
Incidence rates of new cases of lung cancer by sex, 1996-2005.

Lung cancer is defined as one of the major health problems in Thailand and has been the most common cause of death since 1999 [4]. Lung cancer in Thailand is the second most common cancer in males after liver cancer and the fourth in females after cervix, breast and liver cancers. There is a higher incidence rate of lung cancer in northern Thailand than other areas [5]. In 2005, there were 535 new cases of lung cancer diagnosed in 2005 (326 males, 209 females) (Figure 2 and 3) in Northern Thailand. This constituted 25.6% of all cancers in males and 14.9% of those in females. The age-standardised incidence rates were 38.0 for males and 21.7 for females. Lung cancer has ranked first for new male cancers in Chiang Mai since the first population-based registration in 1983 until 2005 in this report. For females, lung cancer ranked third in 2005 after breast and cervix cancers. The incidence rates increased with age in both sexes, with the rates in males increasing sharply after the age of 45 years and exceeding those in females. The cumulative rate percent to age 75 were 4.9% for males and 2.7% for females. The risk of men developing lung cancer by the age of 75 years was 10 in 205 for men and 10 in 376 for women.

**Figure 4:**
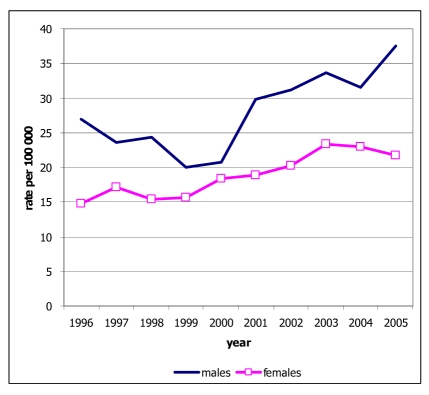
Mortality rate of lung cancer by sex, Chiang Mai, 1996-2005.

**Figure 5:**
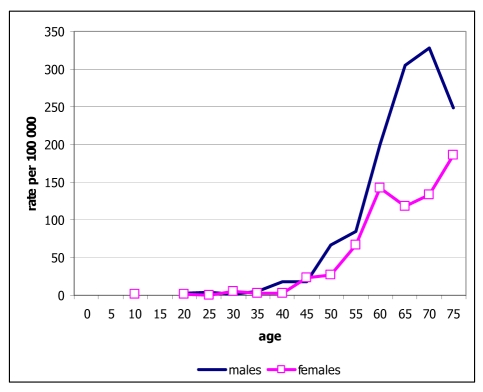
Age-specific mortality rate of lung cancer, Chiang Mai, 2005.

Among the 534 deaths from lung cancer, 327 were males (28.9% of all male cancer deaths) and 207 were females (24.0% of all female cancer deaths). The age-standardised mortality rates were 38.5 for males and 21.9 for females, and these rates were increased in both sexes (Figure 4). The mortality rates increased with age in both sexes, with rates in males increasing sharply after the age of 45 years and exceeding those in females (Figure 5).

Among lung cancer deaths, 375 cases (70.2%) died within one year of diagnosis and 116 cases (21.7%) died in the second year.

### Diagnosis and stage of lung cancer

Fifty percent of cases were diagnosed in advanced stage (36.6% had distant metastasis, 13.1% had regional nodes metastasis). The most common metastasis site was distant lymph nodes, followed by brain (Table 4). One hundred and thirty cases (40.7%) were diagnosed by clinical diagnosis and 85 cases were diagnosed by death certificate only. The common cell types were adenocarcinoma (30.1%) and squamous cell carcinoma (15.7%).

**Table 4:** Histological Cell type and Staging of Lung Cancer

	**males**	**females**	**both**	**%**			**cases**	**%**
Adenocarcinoma	96	65	161	30.1		Localised	23	4.3
Squamous cell	55	29	84	15.7		Locally advanced	148	27.7
Small cell	21	7	28	5.2		Regional node metastasis	70	13.1
Large cell	8	15	23	4.3		Distant metastasis	196	36.6
Others	16	5	21	3.9		Unknown/not stage	98	18.3
Clinical diagnosis	130	88	218	40.7				
**All**	**326**	**209**	**535**			**All**	**535**	

In terms of diagnosis and staging for lung cancer, plain film chest radiography and computed tomography (CT) of the chest and upper abdomen including the liver and adrenal glands are necessary. For endobronchial lesions, pathologic diagnosis can be obtained from bronchoscopic biopsy or CT-guided needle biopsy. Mediastinoscopy and biopsy of mediastinal nodes are commonly performed during preoperative assessment of patients with resectable lung cancer. Pulmonary function tests are utilised to identify patients at high risk of surgical complications. At present, the routine screening for lung cancer in heavy smokers using CT scans is not implemented.

### Treatment of Lung Cancer

Various platinum-based chemotherapy regimens have been utilised in combination with radiotherapy. Cisplatin-based chemotherapy regimens such as cisplatin plus etoposide are commonly used. Other regimens including carboplatin/cisplatin plus paclitaxel, Cisplatin/carboplatin plus gemcitabine and Cisplatin plus vinorelbine have also been utilised.

Palliative thoracic radiotherapy is commonly used for relieving symptoms caused by advanced disease. Various total dose and fraction arrangements were considered for controlling symptoms: 13 Gy given in 2 fractions, or 8 Gy single-fraction, or 20 Gy over 5 fractions are commonly used. Whole brain radiation is usually indicated for stage IV non-small cell lung cancer with intracranial metastases. Prophylactic cranial irradiation is recommended for small cell lung cancer patients with good response from the primary treatment. Palliative radiotherapy for bone pain and prevention of fractures in weight-bearing bones among patients with bone metastasis is commonly used.

### Cervix cancer

There were 234 new cases of cervix cancer diagnosed in 2005. This was 10.3% of all cancers in females. The age-standardised incidence rates were 22.7 and tend to be slightly decreased (Fig 7). Cervix cancer was one of the three most common cancers in females. In 2005, cervix cancer ranked second after breast cancer. The incidence rates increased sharply after the age of 25 and were more common than breast and lung cancers in the age group 15-44 years. The mean age at diagnosis was 50.4 years and the median age at diagnosis was 48 years. The cumulative rate percent to age 75 were 2.3%, representing a 1 in 44 risk for women developing cervix cancer by the age of 75 years.

**Figure 6:**
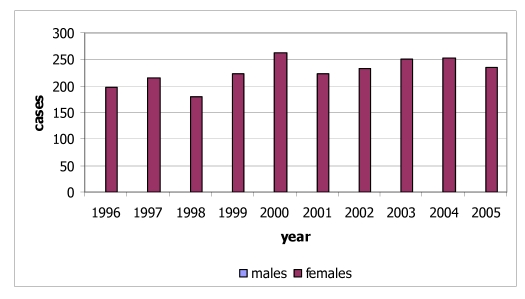
Number of new cases of cervix cancer by sex, 1996-2005.

**Figure 7:**
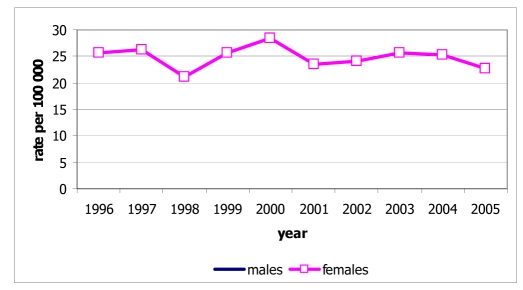
Incidence rates of new cases of cervix cancer by sex, 1996-2005.

Screening for cervical cancer uses the Pap test to detect the presence of premalignant or malignant cells of the uterine cervix. Although human papilloma virus (HPV) is a major risk factor for uterine cervical cancer, testing for HPV deoxyribonucleic acid (DNA) is not a component in the routine screening of cervical cancer in Northern Thailand.

**Figure 8:**
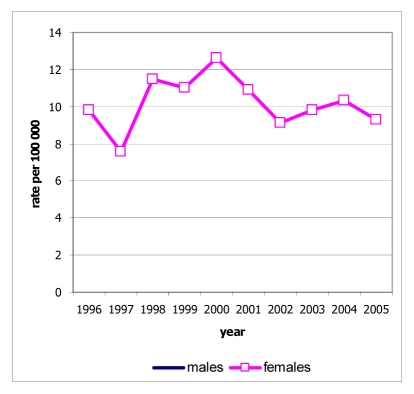
Mortality rate of cervix cancer by sex, Chiang Mai, 1996-2005.

**Figure 9:**
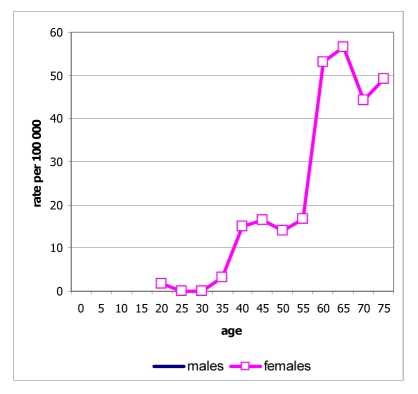
Age-specific mortality rate of cervix cancer, Chiang Mai, 2005.

There were 89 deaths from cervix cancer, accounting for 10.3% of all female cancer deaths. The age-standardised mortality rates were 9.3 and tend to be decreased after 1998 (Fig. 8). The mortality rates increased with age, and increased sharply after the age of 55 years (Fig 9).

Among cervix cancer deaths, 24 cases (27.0%) survived more than five years, 34 cases (38.2%) survived more than three years and 15 cases (16.9%) survived less than one year.

### Diagnosis and stage of cancer

There were 223 cases of carcinoma in situ of cervix uteri which were not included in this analysis. The most widely accepted staging system for cervical cancer is the FIGO (International Federation of Gynecology and Obstetrics) system. Among the invasive cancers, 112 cases (47.9%) were diagnosed in localised stage and 6 cases had distant metastasis. The most common metastasis site was intra-peritoneum seedling. Ninety-eight percent were histologically diagnosed and the common cell types were squamous cell carcinoma (79.1%) and adenocarcinoma (17.1%) (table 5).

**Table 5:** Histological Cell type and Staging of Lung Cancer

**Cell type**	**males**	**females**	**both**	**%**		**Stage**	**cases**	**%**
Squamous cell ca.	-	185	185	79.1		Localised	112	47.9
Adenocarcinoma	-	40	40	17.1		Locally advanced	104	44.4
Others	-	6	6	2.6		Regional node metastasis	6	2.6
Clinical diagnosis	-	3	3	1.3		Distant metastasis	6	2.6
						Unknown/not stage	6	2.6
**All**		**234**	**234**	**100.0**		**All**	**234**	**100.0**

The patient is staged by physical examination including a bimanual pelvic examination with attention to parametria and pelvic side walls, rectovaginal examination, as well as examination of regional lymph nodes. Imaging studies recommended include chest radiography, intravenous pyelography (IVP) and computed tomography (CT). In some situations magnetic resonance imaging (MRI) will help to identify local involvement of the disease, as well as evidence of metastatic spreading. Cystoscopy or proctoscopy is indicated in advanced diseases in which bladder and /or rectal involvement are suspected.

### Treatment of Cervical Cancer

#### Surgery

Surgery alone can be utilised for carcinoma in situ (CIS), stage IA1-2 and IB1. Primary surgery is often chosen for younger patients to preserve ovarian function. Surgery is commonly used in conjunction with chemotherapy and radiation therapy for patients with bulky disease.

#### Radiation Therapy

Radiotherapy alone can also be used for CIS, stage IA1-2 , and IB1 disease but is often chosen for older patients to avoid surgical risks. Radiation can be delivered with external beam and/or intracavitary approaches. Postoperative radiotherapy is recommended in patients whose disease exhibits high-risk features including bulky tumours, lymphovascular invasion, deep cervical stromal invasion, positive lymph nodes, and positive margins. Radiation therapy is delivered with concurrent chemotherapy for patients with stage IB, II, III and IVA cancers.

#### Chemotherapy

Current chemotherapy recommendations are platinum-based regimens. Cisplatin-based chemotherapy concurrent with radiotherapy affords improved locoregional control and survival and is the standard of care in Northern Thailand.

This study referred to only the northern part of Thailand, However, the data and findings are important for the planning and delivery of the most appropriate and effective health services for the regional population. The authors recommended that cancer registry is one of the best methods for handling cancer problems in Thailand in terms of serving and guiding the health care plan.
